# Development MPC for the Grinding Process in SAG Mills Using DEM Investigations on Liner Wear

**DOI:** 10.3390/ma17040795

**Published:** 2024-02-07

**Authors:** Ilia Beloglazov, Vyacheslav Plaschinsky

**Affiliations:** 1 Department of Automation of Technological Processes and Production, Saint Petersburg Mining University, 199106 Saint Petersburg, Russia; 2 Mechanical Engineering Department, Saint Petersburg Mining University, 199106 Saint Petersburg, Russia; plaschinskiy_va@pers.spmi.ru

**Keywords:** abrasive wear, impact wear, mineral grinding, SAG mill, liner wear, numerical modeling, DEM, wear resistance, Archard model, model predictive control

## Abstract

The rapidly developing mining industry poses the urgent problem of increasing the energy efficiency of the operation of basic equipment, such as semi-autogenous grinding (SAG) mills. For this purpose, a large number of studies have been carried out on the establishment of optimal operating parameters of the mill, the development of the design of lifters, the rational selection of their materials, etc. However, the dependence of operating parameters on the properties of the ore, the design of the linings and the wear of lifters has not been sufficiently studied. This work analyzes the process of grinding rock in SAG mill and the wear of lifters. The discrete element method (DEM) was used to simulate the grinding of apatite-nepheline ore in a mill using different types of linings and determining the process parameters. It was found that the liners operating in cascade mode were subjected to impact-abrasive wear, while the liners with the cascade mode of operation were subjected predominantly to abrasive wear. At the same time, the results showed an average 40–50% reduction in linear wear. On the basis of modelling results, the service life of lifters was calculated. It is concluded that the Archard model makes it possible to reproduce with sufficient accuracy the wear processes occurring in the mills, taking into account the physical and mechanical properties of the specified materials. The control system design for the grinding process for SAG mills with the use of modern variable frequency drives (VFD) was developed. With the use of the proposed approach, the model predictive control (MPC) was developed to provide recommendations for controlling the optimum speed of the mill drum rotation.

## 1. Introduction

In modern mineral processing, SAG mills are the main grinding equipment. SAG mills have advantages such as high throughput, high product yields and a high degree of disintegration. These mills have characteristics such as short material residence time, discrete control and suitability for large-scale production compared to other types of mills. Therefore, SAG mills are preferred by large-scale mining and processing plants and are increasingly used in various industries [1,2]. The SAG mill is characterized by low mill drum speed and a high working load, which leads to very high energy consumption during grinding of ore raw materials. It accounts for more than 80% of the power consumption at the plant [3,4].

The rotational speed is an important factor related to the operation and grinding efficiency of the mill. Standard process regimes typically employ constant speed grinding, which consumes a lot of energy, has a large impact on mill liner wear and often has a large amount of material going to regrind. As a consequence, the performance of the process is severely limited. However, with the advent of brand-new control and technologies approaches, the emphasis is gradually shifting to energy saving and extending the continuous operation of equipment. Higher demands are being placed on process stability, grinding optimization and reduction of process maintenance and service time. Thus, an effective and efficient approach to optimize and control of the SAG mill is of great economic significance.

Varying the rotational speed of mills can have a significant impact on the process of grinding materials. Varying the rotational speed allows for controlling the degree of comminution, process efficiency and the quality of the final product. Here are some of the main aspects of varying the rotational speed of SAG mills:

- Degree of Grinding: High ball speeds result in more intense grinding of materials, which can be useful for obtaining a finer structure. Low rotational speeds may be preferred for coarser grinding [5].
- Grinding Time: Increasing the rotational speed of the ball mill can reduce the time required to achieve the desired degree of comminution. However, a speed that is too high can lead to both over-grinding and under-grinding on the one hand, resulting in unnecessary energy loss on the other [6].
- Energy consumption: Increasing the rotational speed of the ball mill requires more energy. It is therefore important to find the optimum speed that provides the desired grinding quality with minimum energy consumption [7].
- Final product: Changing the rotational speed can influence the properties of the final product, such as the particle size, size distribution and material structure [8].

It is important to realize that the optimum mill speed may vary depending on different parameters, e.g., grain size, material properties’ ball loading and process requirements. Before changing the rotational speed, beneficiation studies are performed, evaluating the impact on the process and product quality.

In mineral processing, one of the main items of production costs is the cost of repair and replacement of worn-out working parts of the main equipment [9]. Thus, for example, for the operation of SAG mills. the expenses for reproduction of worn balls, rods and lining elements reach the share of energy costs and sometimes exceed them [10,11]. Such high consumption of materials of beneficiation equipment is explained by their intensive wear against abrasive rocks in the process of crushing and grinding [12,13]. A particularly negative impact on the production process is caused by the wear of mill lining plates, as the reduction in thickness changes their initial geometric profile (plate height, front angle, etc.), respectively, reduces the ability to lift the loaded material to a certain height, i.e., reduces the accumulated energy required to destroy the processed raw material, which, in turn, leads to a sharp decrease in the productivity of the mill [14]. Studies have shown [15] that only a reduction in the front angle of the plate edge without reducing its height can seriously affect the efficiency of the grinding process, so the recommended wear rate at which the lining plate should be replaced is 10–20% by weight [16,17]. The problem of intensive wear is combated by manufacturing lifters from wear-resistant materials [18], applying various types of hardening treatments [19] and applying coatings [20].

The classical approach to study the wear process occurring in a ball mill is to experimentally investigate its operation [21,22]. However, it is not always possible to perform a comprehensive study on the operating equipment due to the associated economic losses and disruption of the workflow continuity. At the same time, computer modeling methods [23,24,25], which have become widespread, can serve as an alternative to full-scale studies, which make it possible to exclude the need to conduct full-scale experiments [26,27,28].

A large number of studies have been devoted to the development of models that would make it possible to determine the parameters of wear occurring under various types of wearing and operating conditions. Among the most famous wear models are the theories of Greenwood and Tripp [29], which take into account the contact interaction between two rough surfaces; the energy theory of Fleischer [30], based on the law of surface fracture; and the theory of Kragelsky [31], which takes into account a large number of factors, so it can be used to describe different types of wear, but this approach is most often used to determine the parameters of fatigue wear. From the point of view of engineering practice, we can distinguish the Archard model [32] used for modeling wear and based on the theory of uneven contact. Archard’s steady-state wear equation has been extended to the running-in mode using a transfer ratio curve representing the initial surface topography [33]. The basic idea of the modern theory is that sliding of microroughnesses of a solid body relative to another body results in compression and tension stresses, which are cyclic in nature and lead to the accumulation of breakage, resulting in wear products.

The discrete element method (DEM), which is based on the Lagrangian approach and considers granular materials as a set of individual interacting particles, each of which is governed by physical laws, is actively used for detailed studies of grinding processes [14,34]. The use of this method makes it possible to predict the kinematic, dynamic and energetic behavior of the loaded material, determining such parameters as normal and tangential components of particle collision forces, impact velocity, sliding distances during their interaction, etc. [35]. Using various software products based on DEM modeling, experts determine the characteristics of the movement of mill media [36], predict the wear of lining elements and grinding bodies [10] and determine the energy consumption under different conditions [37]. 

Recently, an integrated approach has been used for modeling of grinding processes. Zhukovskiy et al. [38] considered the modeling of the grinding process by the joint DEM-CFD method and, on the basis of spectral dependencies, determined the efficiency of the grinding process. Cleary et al. [39] also used spectral dependencies to evaluate the influence of changes in technological variables of the grinding process on the parameters of the electromechanical system. Another example is the use of coupled DEM and SPH models [40], which make it possible to predict the motion of large particles and slurry in a SAG mill.

There is also a large number of works related to the development of digital models [26,41], taking into account the analysis of the electric drive system, which makes it possible to form the basis for the development of integrated methods of the monitoring and evaluation of energy efficiency. This approach allows for the use of digital models for the detection of pre-emergency situations by determining the stress-strain state [42] of individual mill units. 

A fundamental part of advanced process control (APC) systems based on intelligent models of control objects is MPC. The use of the control principle with a predictive model allows for the realization of real-time operational control in optimal modes [42]. An example of implementation is [43], which describes the experience of developing a control system based on MPC on the basis of a neural network, the main tasks of which are to control the target process parameters while minimizing the specific fuel and electricity consumption. In another paper [44], dynamic modeling and multivariate predictive control strategy for a semi-automatic grinding plant are presented. The results show that it is possible to operate the SAG mill in the optimum zone, compensate for unmeasured disturbances and maximize the throughput without violating the operational constraints.

Based on the results obtained in the presented work, the structure of the automatic control system using VSD was developed. The regulation of the mill drum rotation speed, through the use of VSD in various operating conditions, can effectively reduce energy losses, increase productivity and extend the service life of the equipment [45]. However, the relationship between the optimum rotational speed and various process parameters has not received much attention.

As already mentioned, mill rotational speed is an important factor related to mill operation and grinding efficiency. We propose the MPC-based approach controlling the mill drum speed. This model can predict the optimum rotational speed of the ball mill drum depending on the process parameters. For example, Chenet al. [46] proposed an MPC approach based on a deep belief network (DBN). This model enables combined feedback control, reducing the control system overflow and its oscillations caused by inaccurate feedback control. Delaney et al. [47] used various methods to implement predictive analytics techniques as a result the model estimates mass loss with particle evolution and included cumulative damage and destruction. Saldana et al. [48] considered optimization of particle fracture processes using machine learning, which can significantly improve the quality of control.

In this regard, the integrated use of computer modeling methods, together with experimental studies on the lining materials, seems to be a promising way to solve the problem of wear arising in the operation of mills. The presented paper is organized as follows: the analysis of the grinding process and wear is briefly described. Section 2 contains wear process analysis to visualize and understand the processes that occur as a result of impact and abrasive wear of mill liners. Section 3 contains materials and methods, providing the laboratory study of ball mill liner materials, as well as the numerical modeling of the processes occurring in the mill accounting for lifter wear and particle breakage energy. Section 4 presents the results of field experiments and modeling using DEM. Based on the results obtained, the development of the mill speed control concept is presented, as well as MPC in the form of an application. Section 5 discusses the results obtained. In Section 6, conclusions are drawn, giving some ideas for future work.

## 2. Analysis of the Grinding Process and Wear

**Figure 1:**
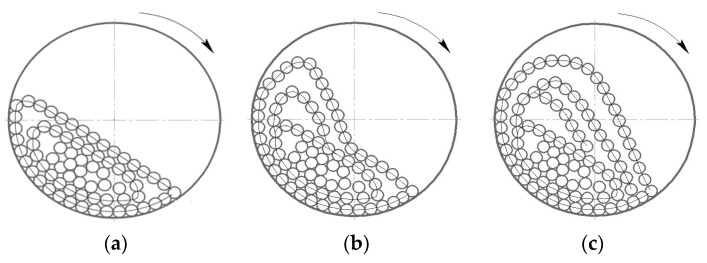
Operating modes of SAG mill: (**a**) cascade; (**b**) mixed; (**c**) waterfall.

The grinding process is determined by the operating mode of the SAG mill, which, in turn, depends on the rotation speed of drum. At a low mill rotation speed (50–60% of the critical speed), the whole ball load makes a turn toward the rotation by some angle, and at a constant rotation speed, it remains in this position. The balls continuously circulate, rising with slippage along concentric circular trajectories and rolling down in parallel layers on other underlying bodies. This mode of mill operation is called a cascade mill (Figure 1a). Grinding of the material occurs mainly by crushing and abrasion by the rolling balls. Cascade mode of mill operation is characterized by fine grinding with increased sludge output and increased wear of the lining due to slippage of the rock-ball loading on the drum wall and, as a consequence, abrasive wear [49]

As the drum rotation speed increases (75–88% of the critical speed), the angle of rotation of the ball load increases and the balls on circular trajectories rise higher, but the mode of operation is still cascade. When, at last, the balls rise to the certain height, determined by the mill rotation frequency, they come down from the circular trajectories and, like bodies thrown at an angle to the horizon, fall back to the circular trajectories along parabolic ones. This mode of mill operation is called waterfall Figure 1c. Grinding of the material in the waterfall mode occurs mainly by impact of the falling balls and partly by grinding. There is also a mixed mode of medium motion (at velocities 60–75% of the critical velocity), shown in Figure 1b, in the part of the grinding bodies rolls down and the other part is in free flight.

According to [49], depending on the operating mode of the mill, the hardness ratio of the lining material and rock, as well as the type of lining, three main types of armor wear can be distinguished.

**Figure 2:**
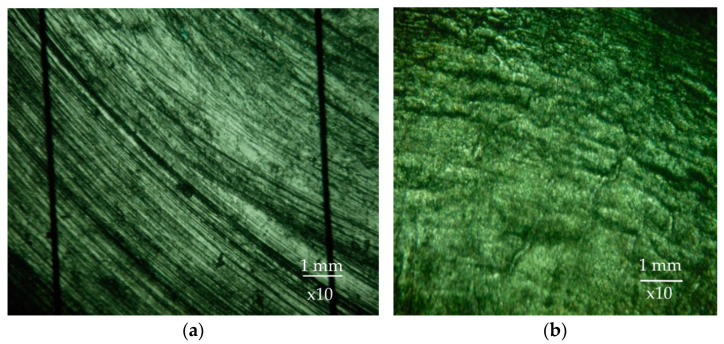
Surface macrostructure of 110G13L steel specimen after wear on granite and gabbro (**a**) and marble (**b**).

In drum mills, abrasive type of wear occurs on smooth and wave liners when processing hard rock, e.g., iron ore with a hardness of 1000 HV in cascade mill operations. Abrasive wear is characterized by the fact that the hard body, called an abrasive, in contact with another, softer one, infiltrates into its surface layer and has the cutting effect when they move relative to each other. In this case, characteristic grooves are formed on the surface layer of the softer body, oriented along the direction of movement of the abrasive particles (Figure 2a).

Contact fatigue wear is observed in the mill at all types of operation modes in the process of grinding relatively soft rocks such as, for example, limestone. The rock particles, due to low stresses, cause elastic rather than plastic deformation of the lining metal. Repeated cyclic contact of the medium with the surface leads to the appearance of a network of microcracks on the surface of the lining. Further microcracks grow deep into the material and lead to the formation of fatigue crack, which causes destruction and removal of metal fragments of the surface layer [50]. A view of the lining is shown in the figure, which shows that the surface is covered with elongated elevations and microcraters (Figure 2b).

**Figure 3:**
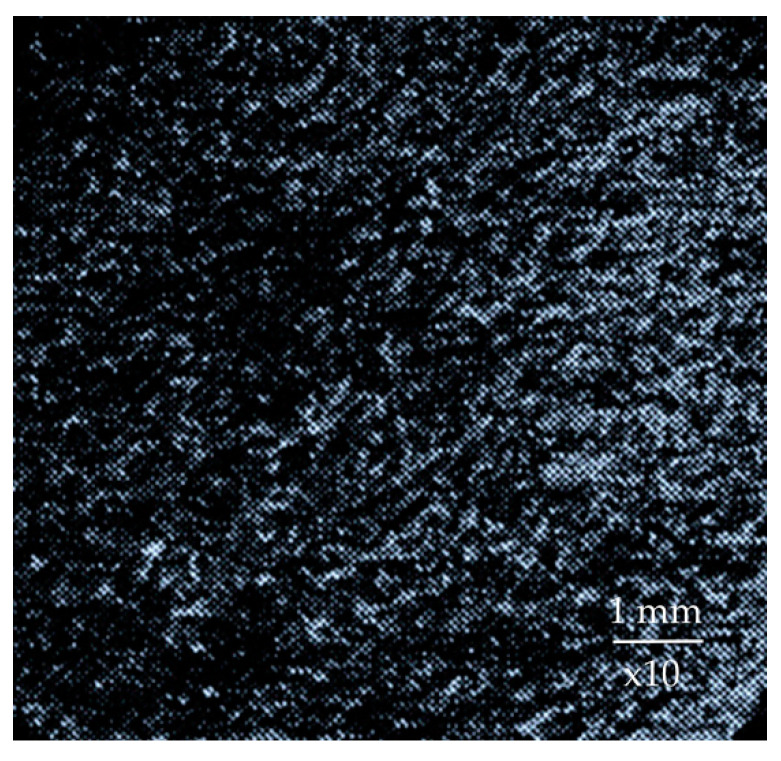
Surface macrostructure of 110G13L steel at impact-abrasive wear.

Impact abrasive wear occurs when grinding rock with the hardness higher than the hardness of the mill lining. This type of wear is caused by impact dynamic penetration of hard abrasive particles into softer material, which causes plastic deformation of the surface layer, which further leads to the destruction of micro-volumes of metal and the formation of holes. The solid particles, once embedded in the wear surface, tend to dislodge the metal of the wells by re-deformation or brittle pitting depending on the hardness of the lining. Accordingly, when grinding the rock harder than the lining material, when the mill operates in waterfall or cascade-waterfall mode without noticeable slippage of the ground rock, the rock grains plastically deform the metal without having a cutting action on it [51]. The surface subjected to impact-abrasive wear has the appearance shown in Figure 3, which shows a combination of holes separated by lintels.

## 3. Methods

### 3.1. Laboratory Study

On the basis of previous studies [49,52], the wear resistance of 110G13L («Petrostal» Metallurgical Plant, Saint Petersburg, Russia) steel was determined in comparison with wear resistance of Hardox (Stockholm, Sweden) (H400, H500) steels of SSAB Oxelosund AB (Stockholm, Sweden) and Miiluks (M450, M500) steels of Miilux Oy (Raahe, Finland) in conditions of their wear by friction on highly abrasive rock. In addition to these steels, the object of tests were carbon steels 45, U8 and 65G and cast irons SCH21 and VCH35, as materials also used for the manufacture of wear elements of mining equipment, including lining plates of mills. Casting steel 25L, widely used in mining engineering, was chosen as a material of comparison. 

The methodology of experiments was as follows: cylindrical specimens with diameter D = 8 mm, height H = 35 mm were made from rolled steel, in the state of delivery, and castings of materials. Half of the samples from steels 45z, U8z and 65Gz were subjected to hardening (heating to 815 °C and holding, then immersion in water) and low tempering at 200 °C. Using a universal hardness tester Zwick/Roell ZHU 187.5 (GmbH & Co. KG, Ulm, Germany), the hardness of the samples was measured by the Rockwell method with further conversion of the obtained units to the Brinell scale. White electrocorundum (25 A) with a grain size of 250–315 microns (250 mg) was used as a homogeneous highly abrasive material with hardness of the main component—Al_2_O_3_ (99.6%, wt.) ~2000 HV, significantly exceeding the hardness of all wear materials.

The analyzed material sample was fixed in the holding device of the unit and pressed with its end surface against the abrasive surface with a constant force of 100 N. Torque was transferred from the electric motor through the spindle and fro the holding device to the sample, providing its movement on the abrasive on a circular trajectory with a radius R = 9 mm with an angular velocity of 7.5 s^−1^. 

In the laboratory study, the wear rate of the material *V_i_* (mg/min) and wear resistance *I_i_* (min/mg), as the inverse of *V_i_*, were determined. The ratio of wear resistance *I_i_* of the material to the wear resistance of the comparison material—steel 25L was taken as the comparative wear resistance *ε* of this steel or cast iron.

### 3.2. Numerical Experiment

To understand the grinding process occurring inside the mill, as well as to obtain numerical values of the kinematic characteristics of the grinding material, an approach for investigating numerical experiments was formulated.

Experimental research, with the help of computer modeling, allows us to effectively study the influence of varying factors on the grinding process in SAG mill. Numerical and laboratory experiments complement each other and allow us to obtain a high accuracy of output data. An important property of numerical experiment is the possibility of visualizing the results of calculations. Presenting the results in a visualized form is the most important condition for their better understanding. In this case, the virtual model allows the researcher to visually demonstrate inaccessible places of the object under study, which is impossible when conducting a real experiment.

In the work, as an object of modeling, we selected mill type MSHTS 5500 × 6500, used for grinding apatite-nepheline ore, with the following technical characteristics: mill capacity Q = 300–310 t/h, drum diameter without lining D = 5500 mm, drum length L = 6500 mm, the working volume of the mill V = 140 m^3^, ball loading m = 275–285 tons, degree of ball loading φ = 42%, lining material—steel 110G13L. The surface profile of the lining plates is wave-shaped; the thickness of the lining plate is 160 mm, and it was 76 mm high. 

According to the technical documentation of mill MSHTS 5500 × 6500 and the type of worn elements of lining, the determining type of wear is impactful and abrasive, occurring at mixed cascade-waterfall mode of operation of the mill at the speed of rotation of the mill drum n = 13.69 rpm.

**Figure 4:**
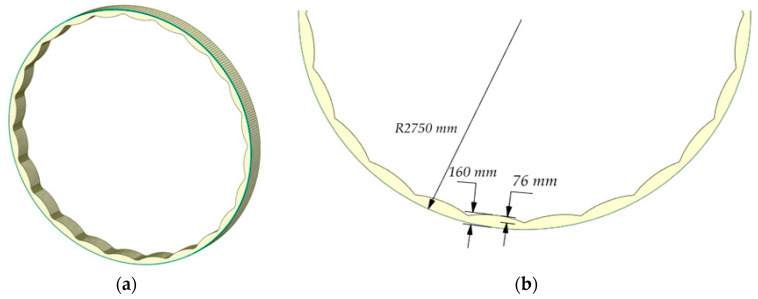
Modeled mill section (**a**) and lining parameters (**b**).

To reduce the calculation time, an object for modeling was taken as a linear section of the cylindrical part of the whole drum 400 mm long (Figure 4). The side walls are zero-friction surfaces in relation to the material and balls. The degree of loading was taken, taking into account the ratio of this section to the width of the whole drum.

**Figure 5:**
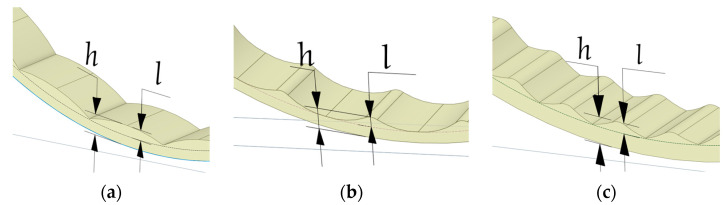
Types of corrugated liners used in modeling: (**a**) type 1; (**b**) type 2; (**c**) type 3.

To obtain a sufficient amount of data for comparison, a series of simulations were carried out for several types of liner profiles parameters of initial liner profiles are presented in Figure 5, where *h* is the height of the entire liner profile, *l* is the height of the wave. For all three types of linings, *h* = 160 mm, *l* = 76 mm.

The finite element mesh size of the geometry was set to 0.1 m, based on the recommendation of having from five to ten mesh elements across the width of the geometry surface as sufficient for accurate wear calculation and adequate presentation of the results [39].

To determine the mill motion, two modes of rotation (motion frame) were set—the first set the constant angular acceleration of 200 rpm^2^ for 3 s to rapid set the mill to the stable mode of operation. After that, the second mode of motion with a constant angular velocity of 13.69 rpm was switched on until the end of the simulation time.

Several approaches have been applied as models of contact interaction. For contacts acting along the normal to the particle surface, the model of the hysteresis linear spring [53] has been applied; for tangential contacts, the Coulomb model of the linear spring has been applied.

Due to the fact that the rock particles are irregularly shaped fragments with a large number of faces, the polyhedrons with 10 vertices were chosen as the shape; for steel balls, the shape of a sphere was set. All other parameters and properties of the medium [54], as well as parameters of contact interactions of the grinding medium–apatite-nepheline ore and grinding material–steel balls, are given in Table 1 and Table 2, taking into account material calibration of material interaction coefficients [35,37].

Based on the degree of ball loading of the mill φ = 42%, we determined the value of ball and rock loading mass for the mill section used in the calculation, which amounted to 15 × 10^3^ kg and 4 × 10^3^ kg, respectively (Table 1).

To determine the wear resistance parameter *K*, we were guided by the fact that it should provide accelerated wear of the geometry compared to the real one. However, it should not lead to rapid and abrupt changes in the geometry, violating the other parameters of the mill operating mode (nature of the medium movement). In practice, the determination of wear resistance parameter is based on calibration from known experimental data, such as the loss of height or mass of liner elements with time [54]. According to the data obtained in production conditions [49], the linings of mill MSHTS 5500 × 6500 are changed at full wear of the wave-shaped lifter profiles, on average, after one year (l = 0.076 m/year).

In Rocky DEM, the loss of material is defined in volumetric units, so linear wear was converted to volumetric. Moreover, taking that the shape of lining is a part of a cylinder, with a radius *r*, then the volume of the element is expressed by the formula: *V =* 1/2*πr*^2^*L*. In accordance with this, the volume of all 24 elements of lining type 1 will be equal to *V* = 0.085 m^3^.

It Is known that the hardness of the material is a mechanical property characterizing the magnitude of the force applied along the normal to the surface for the introduction per unit area. Hence, the hardness of the lining material HB 220 (Brinell scale) can be expressed in units of pressure *H* = 2170 × 10^6^ MPa.

### 3.3. Numerical Methods

#### 3.3.1. Wear Model

Archard model [31] has found its application in describing adhesive and abrasive wear and is used in the Rocky DEM. In this model, wear is defined through the loss of a unit volume of material, which depends on the friction forces generated by the contact of particles with the surface of the material. The volume of worn material is defined as:

(1) $$V=k\frac{F_{t}S_{t}}{H}$$

where *V*—the volume of worn material, m^3^; $F_{t}$—tangential component of the force applied to the surface by the particle at contact action, N; $S_{t}$—distance of sliding of the particle on the surface, m; *H*—hardness of the material, HB; *k*—dimensionless empirical coefficient.

In the Rocky DEM, the surface of objects is divided into elementary triangular sections (facets), with the size set by the user, so the surface wear is realized by the displacement of facet tops inside the object. The distance by which each vertex is moved is calculated to make the volume change equal to the value *dV*, according to the following equation:

(2) $$dV=K\cdot dE_{t}$$

where d*V*—the volume of worn material during the simulation step, m^3^; $dE_{t}$—tangential or shear work performed by particles when colliding with force $F_{t}$ with the surface at distance $S_{t}$ during one time step, J; *K* = *C/H*—wear resistance parameter, selected manually before the calculation that make it possible to set the wear resistance of the material. Based on this parameter, the energy amount used to wear the material in accordance with expression (2) during a time step is determined. Acceleration factor *C* makes it possible to reduce the energy used to wear, since the process of wear modeling, to obtain noticeable results will take a large amount of time. This parameter respectively makes it possible to reduce the simulation time.

To estimate the cumulative energy done by particles in collisions and other parameters, Rocky DEM (ver. 4.3) represents such tools as the module for collecting statistics of collisions of different groups of particles with each other and with boundaries. In addition, it represents the energy spectra module, which takes into account the energy for normal and tangential collisions for each type of particle per time step and for each type of collision. This module also accounts for all combinations of contact pairs: particle–particle, particle–boundary, etc.

In accordance with Equation (2), we found the total energy $E_{t}^{\prime }$ required for the wear of the 24 elements of the lining during its operation for one year (MJ):

(3) $$E_{t}^{\prime }=F_{t}S_{t}=V\cdot H=0.087\times 2.17\times 10^{9}=188.79$$

We used the obtained energy value to determine the wear coefficient in the simulation. The coefficient *K* also takes into account the duration of the simulation process, which needs to be determined to obtain accelerated but uniform wear of the lining, as well as to collect sufficient statistics to evaluate the process parameters. In this regard, 60 s for simulation was taken as the modeling duration. For this purpose, according to Equation (2) in the calculation of the coefficient *K*, we take into account the acceleration factor *C* = 5.3 × 10^5^, which characterizes the specified number of minutes in terms of a year; then, taking this into account, the wear parameter turned out to be equal to *K* = 1 × 10^−5^. The beginning of the collection of the profile wear statistics was set as 5 s after the process setup to stable mode.

Using the energy spectra, the tangential energy of collisions of rock particles with the liner boundary is analyzed, and the dependence of the particle collision power $P_{t}$ on the energy of a single collision is plotted. In addition, the cumulative power of all collisions $P_{ts}$ of rock particles with the lining as a function of collision energy was determined by the formula.

(4) $$P_{ts}=\sum_{j=i}^{n}P_{t}$$

where $P_{t}$—power of collision of rock particles with lining, J/s.

#### 3.3.2. Energy of Particle Breakage

In terms of the efficiency of the grinding process in SAG mills, one of the main problems in finite element modeling is the processing and evaluation of simulation results. As a result of disintegration of the ore, the number of particles increases exponentially, and the numerical modeling time increases accordingly. Therefore, the evaluation of the grinding process is performed using statistical methods that take into account the contact interaction for all contact pairs of interacting materials. In this regard, the so-called energy spectra are used as a tool to analyze the efficiency of the grinding process. 

These spectral dependences of the accumulated impact energy on the specific minimum energy allow us to establish the particle breakage energy for different particle compositions, which, in turn, can be used to select the optimum mill rotation speed and loading.

The average specific energy of particles breakage, with a probability of 50% there will be breakage of the particle for each contact pair, is determined by equation [37]:

(5) $$E_{ave}=\frac{m_{g}\;h\;\;g}{3.6\;m_{p}}$$

where *m_g_*—weight mass, kg; *h*—average drop height at which particle breakage occurs, m; *g*—gravity, m/s^2^; *m_p_*—average mass of particles, kg. 

If we assume that the probability of particle breakage obeys Gauss distribution, then the nominal specific energy of particle destruction will be determined by the formula [37]:

(6) $$E_{m}=E_{ave}+2\sigma$$

where *σ*—the standard deviation of the achieved specific breakage energy. 

It should be taken into account that each grain size class will be characterized by its own average value of nominal specific minimum energy required to breakage the particle. The larger the particle is, the lower the nominal specific breakage energy is. This is due to the natural fracturing of ore raw materials: statistically, in large ore samples, there are more cracks of various degrees of opening than in small ones.

#### 3.3.3. Model Predictive Control

For the predictive model, the relationship between the input and output related to the mill load rate is defined according to the discrete-time equation:

(7) $$\;C\left(\tau \right)=\omega (\rho )\frac{F(\tau )}{R(\tau )}$$

where *τ*—the discrete time; F(τ)—the degree of ball loading in the mill, which is a nonlinear function of material size reduction; t/h, R(τ)—the mill rotation speed, rpm; ω—takes into account the lifter geometry, internal diameter and ball material; ρ, (h·rpm·%)/t.

To avoid the nonlinear formulation of MPC and to assume that the mill control system is not active when the drum is not rotating, Equation (7) can be replaced by a linear one and equated to zero:

(8) $$\;q\left(\tau ,\rho \right)=C^{\prime }\left(\tau \right)\cdot R\left(\tau \right)-\omega \left(\rho \right)\cdot F\left(\tau \right)$$

where *q*—the fictive variable to control the degree of ball loading of the mill with specific breakage energy; *C*′(*τ*)—the current value of the degree of ball loading of the mill.

## 4. Results

### 4.1. Experimental Results

On the basis of laboratory investigation, the values of *V_i_*, *I_i_* and *ε* of analyzed steels and cast irons were obtained. In Table 3, the presented parameters are arranged in the order of increasing wear resistance of materials, as well as the values of their hardness.

**Figure 6:**
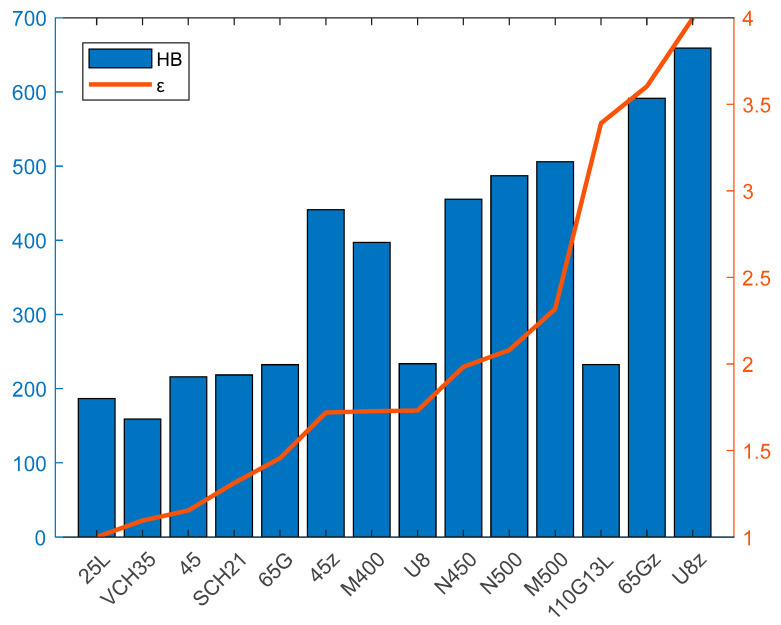
Comparative wear resistance ε and hardness HB of analyzed materials.

The results of the experiment according to Table 3 are shown in the diagram presented in Figure 6, where the comparative coefficients of wear resistance ε of the analyzed materials relative to steel 25L, which is taken as a reference. The materials are arranged in the order of increasing ε in comparison with their hardness.

As follows from the results of experiments in Figure 6, the investigated materials differ significantly in resistance to highly abrasive hard medium: the wear resistance *I_i_* of the most resistant material (steel U8z after hardening) is about four-times higher than this indicator for low-carbon steel 25L, practically not wearing the abrasive surface during wear. In general, as can be concluded from the diagram of Figure 5, the order of arrangement of the tested materials by increasing wear resistance coincides with their arrangement by increasing hardness, which is in accordance with the results generally accepted within previous studies [49]. The steel 110G13L differs from the general order, which, at relatively low hardness (233 HB), shows the highest wear resistance coefficient, significantly exceeding (from 2- to 3.5-times) the value of *I_i_* of all tested carbon steels. It should be noted that the obtained result does not confirm the point of view [20], as steel 110G13L on resistance to purely abrasive wear does not differ from ordinary carbon steel of the same hardness: at the same hardness, steels 65G and U8 steel 110G13L shows significantly higher (about two-times) wear resistance. 

The obtained data on the wear resistance of different materials will be used in the further modeling of the mill lining wear.

### 4.2. Numerical Results

#### 4.2.1. Energy Spectra

Using the energy spectra tool, the Rocky DEM provides data on how energy is transferred between particles in a simulation. The collision data collected in the simulation are classified into three types of collision-related energy: dissipated energy (energy irreversibly converted to other forms during the particle collision), impact energy (normal force energy of the collision) and shear energy (tangential force energy of the collision).

**Figure 7:**
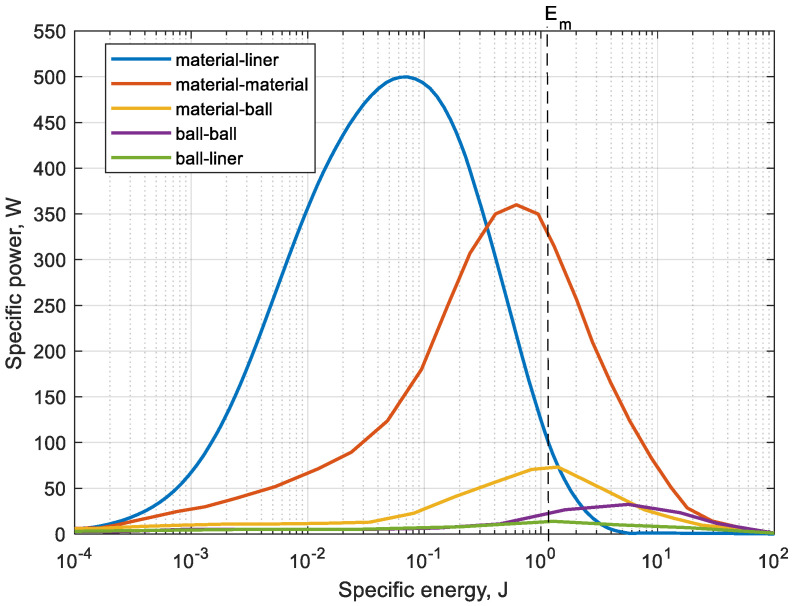
Determination of boundary for particle breakage from the energy spectrums using specific energy at the logarithmic scale.

The obtained spectral energy dependences characterize the ratio of the value of total specific power to specific contact energy on a logarithmic scale. Figure 7 shows the modeled energy spectra for the investigated material and liner type 3.

Figure 7 represents the energy spectra considering the contact interaction for all contact pairs according to Table 3. The value of the nominal specific energy of particle breakage $E_{m}$ in accordance with Equation (6) was determined. This boundary defines two zones in Figure 7. Before the boundary, specific energy is insufficient for particle breakage, but after the boundary, a zone of “useful” collisions is formed, where particle breakage will occur.

**Figure 8:**
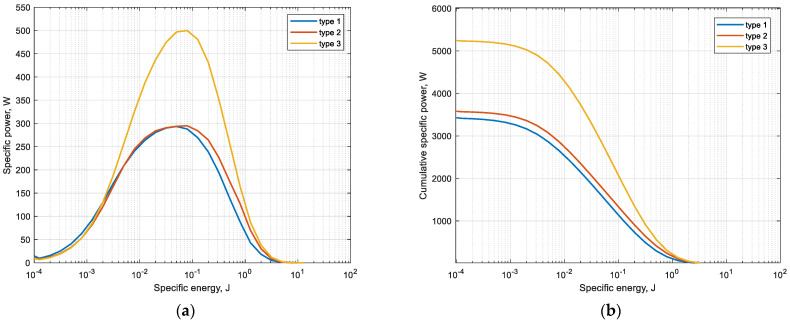
Dependence of specific (**a**) and cumulative (**b**) energy of material-lining collisions.

Thus, having obtained the data of the nominal energy spectra in the whole range of the practical size as given, it is possible to determine the overall efficiency of the grinding process. As the result, energy spectra were obtained for three types of liners (Figure 8a). The cumulative specific power is the sum of the collision powers $P_{t}$ that do and do not cause particle breakage in accordance with Equation (4) (Figure 8b).

In Figure 8, the *x*-axis is represented in logarithmic scale. For this range, values are determined by the minimum and maximum of the shear energy $E_{t}$ of particle collision with the liner boundary. The *y*-axis is equal to the corresponding value of specific power $P_{t}$ generated by all collisions of particles with the lining. 

The presented graph in Figure 8b differs from the previous one in that it also characterizes the cumulative power of all collisions depending on the value of the shear energy $E_{t}$. Therefore, the *y*-axis of the cumulative power is the result of the sum of all points on the power curve. From this figure, it can be noted that, for example, during the simulation of the type 3 lining, the rock particles collide with energies ranging from 1 × 10^−4^ to 1 J. And the maximum cumulative power of all collisions of this range of energies is about 5100 W. Thus, it is possible to draw a conclusion that the greatest share in generation of cumulative power is made by impacts with energy value from 0.01 to 1 J, that also reflects on lining wear.

It is also evident from the Figure 8a,b that linings 1 and 2 show 1.6-times lower values than type 1. This is due to the nature of particle fall, which produces lower kinetic energy of particle fall. 

**Figure 9:**
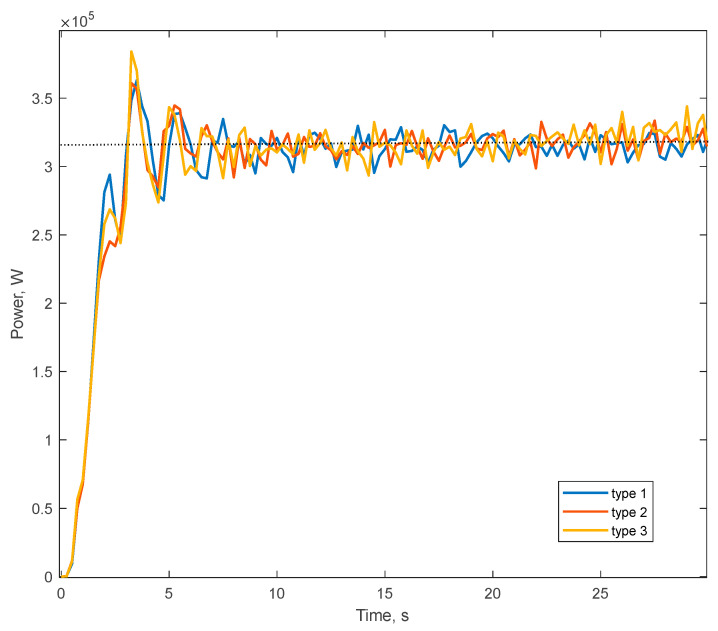
Mill power consumption.

Figure 9 shows the total power consumed by the mill, obtained from the simulation results after the mill setup steady-state operation (after 5 s). It can be noted that for all types of liners, the power value was set approximately at the same level of 3.2 × 10^5^ W.

Recalculating this parameter, taking into account the real length of the mill drum of 6 m, we obtained the average power consumption of 3.25 MW. Taking into account the power losses in the motor main bearings, ring gear, pinion shaft bearings and gearbox, we calculated that the total power consumed by the mill drive is 3.928 MW, which is close in value to the actual power consumption of the mill drive, 4 MW.

#### 4.2.2. Lining Wear Modeling

Table 4 shows the parameters of the analyzed linings after simulation with respect to the mill operating time per year. Based on the difference between the initial size of the surface profile *l* and the one worn during the simulation time, the linear wear of the liner profile ∆*l* was estimated. The volume loss of the liner ∆*V* was also calculated, and then the percentage of the worn surface of the liner *L* from the total size of the liner was determined.

The following parameters were determined: the linear wear of the lining profile ∆*l*, the loss of the lining volume ∆*V* and the percentage of the worn part of the lining from total size.

As can be seen from the data in Table 4, the least amount of wear is demonstrated by corrugated lining 1; on average, linear wear was 50–66% less than that of linings 2 and 3.

**Figure 10:**
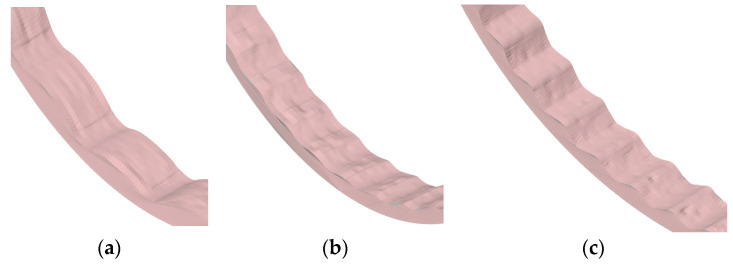
Results of wear modeling: (**a**) type 1; (**b**) type 2; (**c**) type 3.

The simulated liner geometry was imported into the Ansys SpaceClaim (2022R1), and the worn liner profiles were measured. It was assumed that the loss of linear dimensions of the material due to wear could be determined by the change in the height of the lining profile *l*, so the total volumetric loss of material Δ*V* was calculated. The appearance of the worn elements is shown in Figure 10, and the measurement results are shown in Table 4.

As can be seen in Figure 10a, a significant part of the surface of liner 1 contains a large number of grooves characteristic of abrasive wear, which corresponds well with the type of worn surface in abrasive wear. On the surfaces of linings 2 and 3 (Figure 10b,c), except furrows, it is possible to also see pits characteristic for impact wear; their quantity increases at the transition to lining 3, which allows us to conclude that the type of wear at linings 2 and 3 is impact-abrasive, corresponding to the image of the given type of wear in Figure 2.

**Figure 11:**
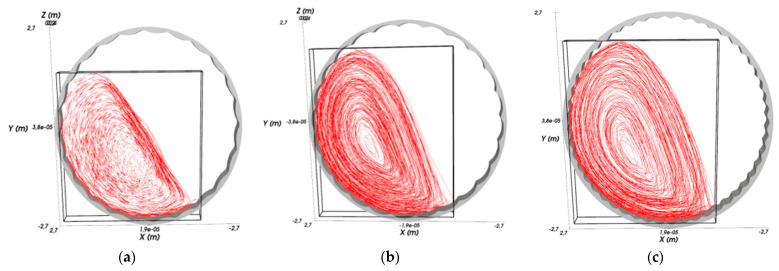
Trajectory of the medium: (**a**) type 1; (**b**) type 2; (**c**) type 3.

**Figure 12:**
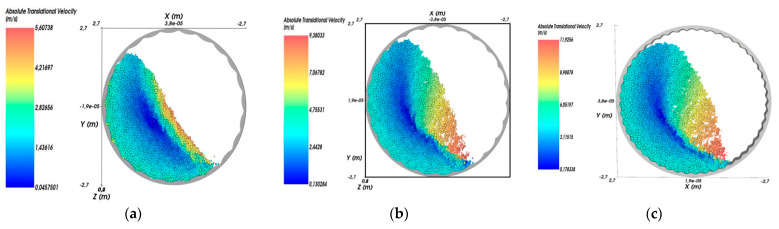
Medium velocity distribution: (**a**) type 1; (**b**) type 2; (**c**) type 3.

Figure 11 and Figure 12 show simulation results for the trajectory of the medium in the mill and the distribution of the velocity of the medium particles.

In Figure 11, the red lines indicate the trajectories of the media during the drum rotation. Liner type 1 (Figure 11a) is represented by circular lines at the beginning of the movement and parallel lines at an angle to the horizon after reaching the top point; (Figure 11b) liner type 2 is represented at the beginning of the movement where the trajectory changes from circular to parabolic; and (Figure 11c) with liner type 3, the parabolic trajectories have a gentler slope in contrast to the previous one.

Figure 12 shows the distribution of material velocity in the mill, for each particle, using different types of linings. These figures clearly show the dead zones (blue) and the particles with high velocity (red).

As can be deduced from Figure 11 and Figure 12, in the mill with liner 1 at given operating parameters of the process, the cascade mode of operation occurs, characterized by the fact that the outer layers of the feed medium rise on circular trajectories upward and then roll down in parallel layers (Figure 11a). This mode provides an average velocity of particles falling on the liner within v = 5 m/s (Figure 12a). At the same time, corrugated liners 2 and 3 provide waterfall mode of operation, in which the outer layers of rock and balls move to the upper point of ascent (Figure 11b,c), after which they fall at an angle on the crushed medium and liner (Figure 12b,c), which provides higher values of particle fall velocity on the liner (v = 10–12 m/s) compared to the waterfall mode.

**Figure 13:**
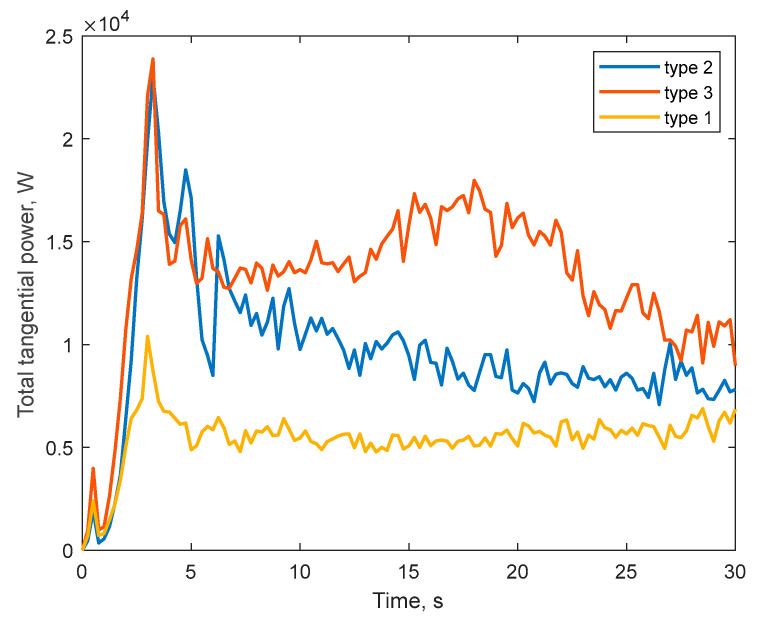
Time dependence of the total tangential power of particle collisions.

Figure 13 shows the dependences of the power $P_{t}$, which is defined through the ratio of the energy $E_{t}$ generated from the tangential components of collisions of all particles with the liner to the simulation time.

As can be seen from the Figure 13, the total specific power $P_{t}$ is set at a certain level of values after the mill setup the steady-state mode of operation (after 5 s). It can be noted that the mill with corrugated lining type 1 in the cascade mode of operation demonstrates the lowest $P_{t}$ value within 6 kW; at the same time, linings 2 and 3, due to the waterfall mode of operation, provide, respectively, 1.66- and 2.5-times higher values of power $P_{t}$, which determines and higher values of the value of wear of these linings at the same duration of simulation.

Based on the results of modeling, we determined the complete wear time, during which the studied liners will completely lose the dimensions of their geometric profile. This approach allows us to estimate their operating time $t_{ot}$ under the given modeling parameters.

For this purpose, we took the ratio of the initial volume *V* of the liners to the volume loss Δ*V* after modeling (Table 4). In view of the fact that the established simulation duration of 60 s corresponds to one year of mill operation, the value of $t_{ot}$ allows us to estimate the wear time of the useful volume *V* during the simulation process. As a result of the calculation, liner 3, which has the largest amount of material volume loss due to wear, will last for 1.12 year ($t_{ot}=V/\Delta V$) or 9811 h. Accordingly, the operating time of lining 2 is 10,440 h, and that of lining 1 is 14,049 h.

We also conducted a series of simulations with duration of 60 s with type 3 of corrugated lining for several steel grades having different hardness and wear resistance *V_i_*, defined by Bolobov et al. [18] (Table 2). According to the previously described methodology, the parameters *E_t_* and *K* were determined for each material. According to the value of wear obtained as a result of modeling, the wear resistance index *V_m_* was determined. Based on the results of Table 5, it can be concluded that it has close values with the index *V_i_*, determined experimentally.

The table shows that the wear resistance *V_m_* of all materials, including Hadfield steel, as well as in physical experiments, increases with increasing hardness, but in the Rocky DEM, the latter does not demonstrate the high property of wear resistance observed in experimental conditions, due to the impossibility of setting some of its physical and mechanical properties responsible for its self-hardening in the wear process.

### 4.3. Model of Predictive Control

**Figure 14:**
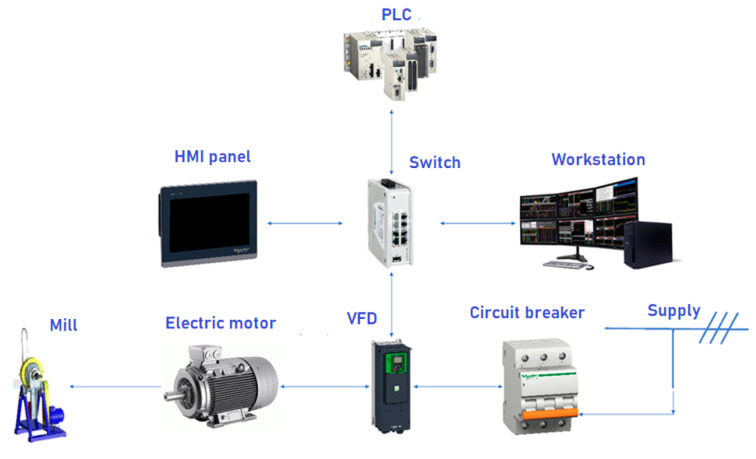
Design of the control system.

The performance of the MPC is based on the development of the control system architecture using VFD and the analysis of energy measurements of the mill electric drive. MPC development is especially relevant for the mining and metallurgical industry [55,56,57]. The frequency converter used in the control system is equipped with scalar and vector control systems, which can operate in sensorless mode and with speed feedback. The general structure is shown in Figure 14.

Schneider Electric M340 (Schneider Electric, «ElectroMonoblock», Russia, Saint-Petersburg) was used as a PLC; however, this controller does not have a dustproof version and therefore requires mounting in a cabinet. For this purpose, cabinet NSYCRN46250P was selected, with IP65 dustproof class and mounting plate included. On the front panel, a Magelis operator panel and an emergency button for emergency stop were mounted.

The Magelis HMIST6500 (Schneider Electric, Taiwan, Taipei) operator panel has a 10-inch display with acceptable resolution and can be hermetically mounted on the control cabinet door without additional means.

The ConneXium TCSESSU083FN0 (Schneider Electric, Germany, Ratingen) switch is required to connect to any external network without additional configuration, due to the fact that the switch is unmanaged.

The frequency drive is connected to the PLC via Ethernet IP protocol, the operator panel is connected via Modbus TCP/IP and the general architecture of the connection is shown in the structural diagram in Figure 13.

**Figure 15:**
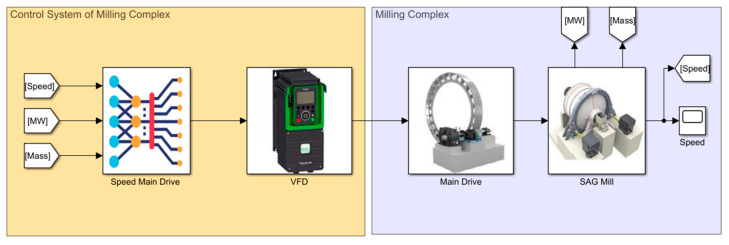
MPC design.

As a basic MPC, we proposed to use the static model of the process developed in MATLAB, taking into account the energy spectra obtained during numerical simulation [58]. The block diagram of the general model in MATLAB Simulink(R2021b) is shown in Figure 15. MPC operation is based on the comparison of the actual power of the mill electric drive (MW), actual speed (Speed), ball load (Mass) and the calculated data obtained on the model. 

The left part of the model in Figure 15 represents the mill control system, which includes the subsystem for calculating the optimal rotational speed (Speed Main Drive) and VFD simulation model. The right part of the system contains a model of the SAG mill with an electric drive [50,51]. For modeling of the electric drive (Main Drive), an asynchronous electric motor from SimPowerSystems Matlab Simulink library was used. The rated power of the Main Drive motor is 4000 kW, Stator voltage is 6 kV, stator current is 447 A and rated speed is 150 rpm. The asynchronous motor is powered by the frequency converter using a Universal Bridge unit controlled by the pulse width modulated signal generating unit (Simulink PWM Generator). The intensity source (Chirp Signal) generates the three-phase signal from the initial frequency to the final frequency according to the law U/f = const. It is possible to generate both increasing and decreasing of the signal according to the specified law.

**Figure 16:**
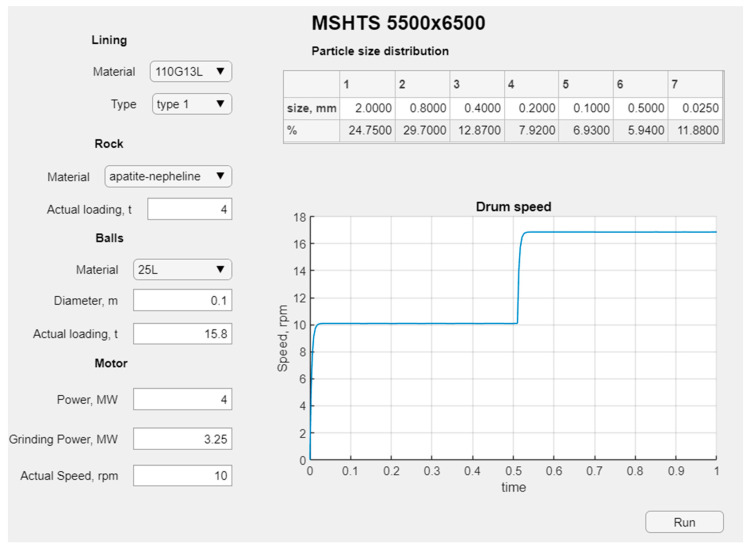
Program interface.

The predictive model, at the current stage of development, runs discretely and requires an input of initial data. This assumption is based on the fact that additional sensors need to be installed on the actual mill to operate in dynamic mode [59]. The operator screen is shown in Figure 16. 

Before commencing the grinding of the material, the material properties, particle size distribution and mass of the material feed and the grinding ball parameters, such as, e.g., the ball diameter and initial ball load, are set on the operator’s screen, as shown in Figure 16. This is necessary in order to correctly initialize the model to be able to optimally select the mill drum speed. The influence of ball loading is one of the main factors in controlling the rotational speed. 

The mill drum speed is maintained in waterfall mode at the initial stage by means of a regulator. Later on, after specifying the material size distribution, it is switched to mixed mode.

**Figure 17:**
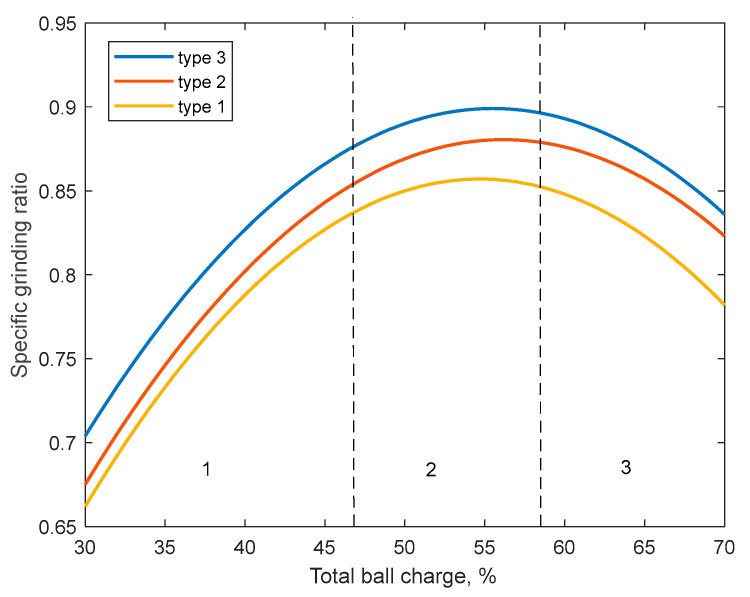
Dependence of grinding speed on total ball loading: 1—underloading zone; 2—optimal loading zone 3—overloading zone.

The ball loading of a SAG mill can be considered optimal if it provides a given finished grade capacity with consideration of minimum energy consumption. Insufficient ball filling most often leads to a decrease in the efficiency of operation on the output of the finished class, and its excess leads to an increase in the power consumption of the mill. On the basis of the developed model, the dependences of the change in grinding ratio on ball loading for different linings are shown in Figure 17.

Thus, the optimum ball loading will correspond to zone 2 in Figure 17. In this case, the zone boundaries are determined based on the material and ball loading. Therefore, the control task is to find the criterion corresponding to the ball loading in the optimal zone (Figure 17). This criterion is the nominal specific energy according to Equation (6).

After, the loading of balls and subsequent grinding of material are carried out. Grinding of the material takes place at constant control of the mill drum rotation speed by means of synchronization with the model. In this case by means of the model the size of the material at the given moment of time is specified, compared with the set size by means of entering the set point from the operator panel, and the data of the rotation speed of the mill drum (Speed) are transfer to the controller (Speed Main Drive), as shown in Figure 15. This ensures the selection of the most optimal speed of rotation of the mill drum, for a given moment in time, depending on the coarseness of the material at the current stage. At the realization of this algorithm on the controller, the table of vectors of speed for VSD is formed. The frequency-controlled drive, in turn, regulates the rotation speed of the mill by changing the frequency of the input voltage to the Main Mill Drive (Figure 15) [36,38].

**Figure 18:**
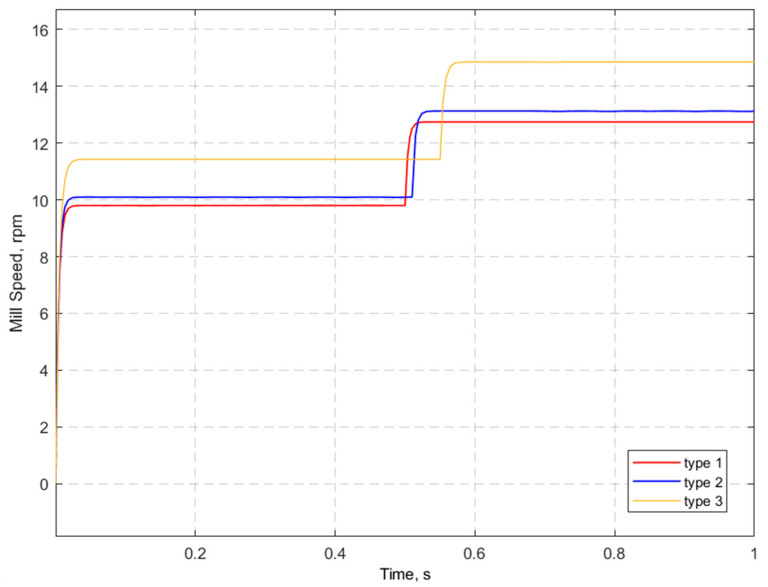
Control system output.

The operation of the program model is demonstrated by the following example. Apatite-nepheline ore is used as feedstock for grinding. In the model, the process parameters, such as the lining type, ore material, initial rotational speed, current mill power, etc., are set (see Figure 16). In the future, the type of lining was changed with unchanged initial parameters. The modeling results for the three types of liners are shown in one Figure 18.

As can be seen from Figure 18, at the beginning, the mill speed is calculated with the initial conditions. The initial estimated motor speed is about 10 rpm for the first two liner types and 11 rpm for the third type. It can be seen that when the time and speed of the motor is zero, it reaches the target speed in the short period of time and tends to be stable. After the lag time, the speed of rotation is recalculated considering the determination of the maximum of the grinding speed function. The lag time in this case is added artificially but can actually vary depending on the transient time. After recalculation, the rotational speeds for the three types of liners are 12.74, 13.12 and 14.86 rpm, respectively. 

During simulation in Rocky DEM at the obtained rotational speeds and liner types, it was found that material movement occurred in waterfall mode (Figure 12c). After further specification of the particle size distribution (reduction of large fraction) in MPC, the rotational velocity decreased, and as a result, the transition to the cascade mode took place (Figure 12a). Recalculation will also occur when other parameters are adjusted.

No matter what the current mill speed was, as can be seen in Figure 17, the target speed was achieved in the fraction of a second and tended to be stable using the developed MPC. It can be concluded that the MPC in this study is suitable for SAG mill motor speed control and can be used both to give recommendations to the operator and add-on of the control system.

## 5. Discussion

It is established that at the same operating modes, depending on the geometric profile of the lining, different modes of medium movement occur. Thus, lining 1 provides the occurrence of cascade mode of operation of the mill, in which there was mainly abrasive wear of the surface. In mills with linings 2 and 3 a waterfall mode with predominantly impact abrasive wear occurred. 

The surface wear index was minimal in lining 1; it was 50 and 66% less than in linings 2 and 3, respectively. This circumstance is explained by the fact that, in the mill with lining 1 at the waterfall mode of medium movement, much less power was generated from tangential interaction of rock particles and balls with the lining than in the mills with lining 2 and 3 ($P_{t}$ was 1.6 and 2.5 less, respectively).

On the basis of the determined value of Pt the estimated service life of linings, t was calculated, which amounted to 7884, 5042 and 4042 h for three linings, respectively.

In the presented work, the interrelation of physical–mechanical parameters characterizing wear resistance and hardness is established, which, in the future, will make it possible to reduce the laboratory tests necessary for the selection of lining materials and significantly reduce the time of simulation modeling. However, setting the value of wear intensity, taking into account the experimentally determined wear resistance parameters, is inexpedient for those materials that are capable of acquiring significant self-hardening, such as, for example, Gadfield steel, due to the impossibility of setting physical and mechanical properties in the program. 

In addition, the proposed methodology will make it possible to select linings and coatings used in the grinding process depending on the properties of the ore. In this case, the grinding process can be carried out using both different types of mills and different types of linings. This will allow for the prediction of the life cycle of composite materials use in the grinding process.

It should also be noted that the profile of the lining will have a significant impact on the character of the medium movement in the drum mill. In this regard, when taking into account the wear of the lining, it is necessary to take into account not only the properties of the material, but also the dynamic nature of the material and ball movement. As the linings wear, the drum volume and, consequently, the useful capacity increases proportionally. However, the experience of SAG mills operation shows that the general technical and economic indicators of grinding with the use of thin linings are worse than those of mills with the thickest lining. As process modeling has shown, it is necessary to increase the coefficient of adhesion of the lining with the crushing medium to enhance the impact and to reduce it to intensify grinding. Further research in the field of modeling of grinding processes in drum mills should be related to the shape of liners and the nature of their wear.

The more precise determination of the grinding process parameters (for example, the weight sensor or sensor for precise measurement of toe position [60]) will, in turn, allow for the real-time monitoring of the loading to prevent damage to the liners and to improve control and optimize mill operation. This additional parameter can also be used to calculate the total mill and ball load. Morrell’s C-model [61], which describes the geometry of the charge using different sub-equations, can be used for modeling. Determining the toe position will allow for an accurate calculation of the total mill charge, taking into account the mill speed [62,63].

It is important to note that a combination of different models can be used to determine the total mill SAG charge or rotational speed using predictive models. The different methods can be used individually, but if two or more of them are used together, it will provide a validation of the individual methods.

Grinding models are based on certain and measurements that may contain uncertainties. The use of multiple methods provides a mechanism to verify that the results are consistent. Any discrepancies between the values obtained indicate a problem, such as poor sensor calibration, or possibly an early warning of abnormal liner wear. The proposed predictive model will allow to improve the measurement, control and ultimately the optimization of SAG mills. Moreover, MPC can be implemented in dynamic mode after refinement for the digital twin approach implemented.

The performance and energy efficiency of a SAG mill can be improved by optimizing aspects such as the liner, rotational speed and total mill feed. A mill fill that is too low can reduce energy efficiency and increase liner wear; a fill that is too high can reduce performance and eventually lead to the mill overloading. However, mill load measurements such as strain gauges or bearing pressures also take into account the mass of the mill and linings, which changes with wear. Therefore, the use of predictive models will allow adjustments to be made to the control system without stopping and inspecting for process maintenance.

## 6. Conclusions

The SAG mill model was created using a discrete element simulation program based on experimental studies of material properties considering abrasive-impact wear. In this work, a complete research cycle, including experimental material investigation, numerical modeling and development of the final software application, was performed. Modeling of the grinding process allowed to identify key features of the process related to energy spectra. The following conclusions can be drawn as a result of this research:

1. The choice of liner type and material has a significant influence on the grinding process in SAG mills. In the process of lining wear, the character of the medium movement will change, and, as a consequence, the material grinding rate and the overall energy efficiency of the process will also change. In addition, the process data obtained will allow for rational planning and longer maintenance intervals in the future.
2. The mill speed control during the grinding process by means of a frequency drive will be the most rational solution. In the case of the developed control strategy, changing the speed increases the grinding performance, as the rise and subsequent fall of ore increases, provided that the mill does not run to the critical speed.
3. The model proposed in this manuscript for determining the optimum speed can be used both as an advisor to the process operator and as part of the control system. It should be noted that the software application is not universal and should be customized for each mill, taking into account their properties and parameters.

Further research can be directed to the study of the grinding kinetics for the feed material and improvement of MPC for operation in dynamic mode. In this way, the mill speed control will compensate for changes in ore hardness. In the control system, this is another disturbing effect. Softer ore requires a reduction in mill speed to maintain and grinding conditions, while protecting the mill lining from increased wear. Conversely, for hard ore, the mill speed is increased to impart more energy to the process.

## 7. Patents

Method of controlling the process of material grinding in a drum mill, I.I. Beloglazov et al. Available online: https://new.fips.ru/registers-doc-view/fips_servlet?DB=EVM&DocNumber=2022683673&TypeFile=html (accessed on 23 December 2023).

Drum Ball Mill Control Program for Schneider Electric M340 PLC and ATV950 Frequency Drive, Beloglazov I.I. et al. Available online: https://www.fips.ru/iiss/document.xhtml?faces-redirect=true&id=2ff8731dfef7ac0e1cd4a4f832b06f2e (accessed on 23 December 2023).

## Figures and Tables

**Table 1:** Simulated material and geometry parameters.

Property	Material	Balls	Lining
Density, kg/m^3^	3.2 × 10^3^	7.7 × 10^3^	7.7 × 10^3^
Young’s modulus, N/m	1 × 10^8^	2 × 10^11^	2 × 10^11^
Rolling resistance	0.15	0.001	0.05
Poisson’s ratio	0.25	0.3	0.3
Fraction size, m	0.18–50%	0.1	-
	0.06–30%		
	0.03–20%		
Ball loading and material, kg	4 × 10^3^	15.8 × 10^3^	-

**Table 2:** Contact pair interaction parameters.

Interaction Parameter	Material–Material	Material–Balls	Material–Lining	Balls–Lining	Balls–Balls
Friction coefficient	0.87	0.5	0.3	0.15	0.15
Restitution coefficient	0.5	0.5	0.3	0.15	0.5

**Table 3:** Parameters of the analyzed materials in comparison with their hardness.

Material	*V_i_*, g/min	*I_i_*, min/g	ε	HB
25L	0.53	1.90	1.0	186.8
BCH35	0.48	2.08	1.1	159.1
45	0.46	2.19	1.2	216.1
SCH21	0.40	2.49	1.3	218.6
65G	0.36	2.76	1.5	232.4
45z	0.31	3.26	1.7	441.3
M400	0.31	3.27	1.7	397.0
U8	0.30	3.28	1.7	233.8
N450	0.27	3.76	2.0	455.4
N500	0.25	3.94	2.1	486.9
M500	0.23	4.39	2.3	506.0
110G13L	0.16	6.43	3.4	232.5
65Gz	0.15	6.83	3.6	591.5
U8z	0.13	7.58	4.0	659.0

**Table 4:** Lining parameters.

Type of Lining	Linear Size of Lining Protrusion *L*, m	Linear Wear of Lining Profile ∆*l*, m	Total Volume of Lining *V*, m^3^	Volume after Modeling *V*, m^3^	Loss of Lining Volume Δ*V*, m^3^	Percentage of Worn Surface *L, %*
*K* = 1 × 10^−5^						
type 1	0.076	0.03	0.085	0.032	0.053	39
type 2	0.076	0.045	0.087	0.014	0.073	59
type 3	0.076	0.05	0.092	0.010	0.082	65

**Table 5:** Comparison of analytical and experimental indicators.

Steel	HB	*K*	*E_t_*, MJ	*V_i_*, g/min	*V_m_*, g/min
45L	200	1 × 10^−6^	174.00	0.46	0.84
110G13L	235	2.5 × 10^−6^	204.45	0.16	0.75
H450	450	7 × 10^−6^	391.50	0.27	0.44

## Data Availability

Data are contained within the article.

## References

[1] Pelevin A.E. (2022). Iron ore beneficiation technologies in Russia and ways to improve their efficiency. J. Min. Inst..

[2] Litvinenko V.S., Petrov E.I., Vasilevskaya D.V., Yakovenko A.V., Naumov I.A., Ratnikov M.A. (2023). Assessment of the role of the state in the management of mineral resources. J. Min. Inst..

[3] Aleksandrova T., Nikolaeva N., Afanasova A., Romashev A., Kuznetsov V. (2023). Justification for Criteria for Evaluating Activation and Destruction Processes of Complex Ores. Minerals.

[4] Morrell S. (2022). Helping to reduce mining industry carbon emissions: A step-by-step guide to sizing and selection of energy efficient high pressure grinding rolls circuits. Miner. Eng..

[5] Bian X., Wang G., Wang H., Wang S., Lv W. (2017). Effect of lifters and mill speed on particle behaviour, torque, and power consumption of a tumbling ball mill: Experimental study and DEM simulation. Miner. Eng..

[6] Bardinas J.P., Aldrich C., Napier L.F.A. (2018). Predicting the Operating States of Grinding Circuits by Use of Recurrence Texture Analysis of Time Series Data. Processes.

[7] Avalos S., Kracht W., Ortiz J.M. (2020). Machine Learning and Deep Learning Methods in Mining Operations: A Data-Driven SAG Mill Energy Consumption Prediction Application. Min. Metall. Explor..

[8] Steyn C.W., Sandrock C. (2013). Benefits of optimisation and model predictive control on a fully autogenous mill with variable speed. Miner. Eng..

[9] Cleary P.W., Morrison R.D., Delaney G.W. (2018). Incremental damage and particle size reduction in a pilot SAG mill: DEM breakage method extension and validation. Miner. Eng..

[10] Hasankhoei A.R., Maleki-Moghaddam M., Haji-Zadeh A., Barzgar M.E., Banisi S. (2019). On dry SAG mills end liners: Physical modeling, DEM-based characterization and industrial outcomes of a new design. Miner. Eng..

[11] Imaichi K., Nordell L.K., Porter B., Potapov A. (2017). Development and performance variation of energy saving type comminution machine using general purpose DEM simulation soft “rOCKY. ” J. Soc. Powder Technol. Japan.

[12] Cleary P.W., Owen P. (2019). Effect of operating condition changes on the collisional environment in a SAG mill. Miner. Eng..

[13] Faria P.M.C., Rajamani R.K., Tavares L.M. (2019). Optimization of Solids Concentration in Iron Ore Ball Milling through Modeling and Simulation. Minerals.

[14] Cundall P.A., Strack O.D.L. (1979). A discrete numerical model for granular assemblies. Geotechnique.

[15] Owen P., Cleary P.W. (2015). The relationship between charge shape characteristics and fill level and lifter height for a SAG mill. Miner. Eng..

[16] Yin Z., Peng Y., Li T., Wu G. (2018). DEM Investigation of Mill Speed and Lifter Face Angle on Charge Behavior in Ball Mills. IOP Conf. Ser. Mater. Sci. Eng..

[17] Yin Z., Peng Y., Li T., Zhu Z., Yu Z., Wu G. (2019). Effect of the operating parameter and grinding media on the wear properties of lifter in ball mills. Proc. Inst. Mech. Eng. Part J J. Eng. Tribol..

[18] Bolobov V.I., Latipov I.U., Zhukov V.S., Popov G.G. (2023). Using the Magnetic Anisotropy Method to Determine Hydrogenated Sections of a Steel Pipeline. Energies.

[19] Hu Q., Ji D., Shen M., Zhuang H., Yao H., Zhao H., Guo H., Zhang Y. (2022). Three-Body Abrasive Wear Behavior of WC-10Cr3C2-12Ni Coating for Ball Mill Liner Application. Materials.

[20] Wu W., Che H., Hao Q. (2020). Research on Non-Uniform Wear of Liner in SAG Mill. Processes.

[21] Liu Z., Wang G., Guan W., Guo J., Sun G., Chen Z. (2022). Research on performance of a laboratory-scale SAG mill based on DEM-EMBD. Powder Technol..

[22] Cleary P.W., Hilton J.E., Sinnott M.D. (2017). Modelling of industrial particle and multiphase flows. Powder Technol..

[23] Cleary P.W., Owen P. (2018). Development of models relating charge shape and power draw to SAG mill operating parameters and their use in devising mill operating strategies to account for liner wear. Miner. Eng..

[24] Gizatullin R., Dvoynikov M., Romanova N., Nikitin V. (2023). Drilling in Gas Hydrates: Managing Gas Appearance Risks. Energies.

[25] Kozhubaev Y., Belyaev V., Murashov Y., Prokofev O. (2023). Controlling of Unmanned Underwater Vehicles Using the Dynamic Planning of Symmetric Trajectory Based on Machine Learning for Marine Resources Exploration. Symmetry.

[26] Rogachev M.K., Aleksandrov A.N. (2021). Justification of a comprehensive technology for preventing the formation of asphalt-resin-paraffin deposits during the production of highly paraffinic oil by electric submersible pumps from multiformation deposits. J. Min. Inst..

[27] Martynov S.A., Pervukhin D.A. (2023). Algorithm for calculating of the carbon-graphite electrode consumption in an ore-thermal furnace and its position at different stages of smelting. Chernye Met..

[28] Greenwood J.A., Tripp J.H. (1970). The Contact of Two Nominally Flat Rough Surfaces. Proc. Inst. Mech. Eng..

[29] Fleischer G. (1973). Energetische Methode der Bestimmung des Verschleisses. Schmierungstechnik.

[30] Kragelsky I.V., Dobychin M.N., Kombalov V.S. (1982). Friction and Wear.

[31] Archard J.F. (1953). Contact and rubbing of flat surfaces. J. Appl. Phys..

[32] Varenberg M. (2022). Adjusting for Running-in: Extension of the Archard Wear Equation. Tribol. Lett..

[33] Morrison R.D., Cleary P.W. (2004). Using DEM to model ore breakage within a pilot scale SAG mill. Miner. Eng..

[34] Bolobov V.I., Chupin S.A., Le-Thanh B. (2022). Modeling impact fracture of rock by hydraulic hammer pick with regard to its bluntness. Eurasian Min..

[35] Boikov A., Savelev R., Payor V., Potapov A. (2021). Universal Approach for DEM Parameters Calibration of Bulk Materials. Symmetry.

[36] Weerasekara N.S., Liu L.X., Powell M.S. (2016). Estimating energy in grinding using DEM modelling. Miner. Eng..

[37] Lvov V., Chitalov L. (2021). Semi-autogenous wet grinding modeling with cfd-dem. Minerals.

[38] Zhukovskiy Y.L., Korolev N.A., Malkova Y.M. (2022). Monitoring of grinding condition in drum mills based on resulting shaft torque. J. Min. Inst..

[39] Cleary P.W., Morrison R.D., Sinnott M.D. (2020). Prediction of slurry grinding due to media and coarse rock interactions in a 3D pilot SAG mill using a coupled DEM + SPH model. Miner. Eng..

[40] Vasilyeva N., Golyshevskaia U., Sniatkova A. (2023). Modeling and Improving the Efficiency of Crushing Equipment. Symmetry.

[41] Goldobina L.A., Demenkov P.A., Trushko O.V. (2019). Ensuring the safety of construction works during the erection of buildings and structures. J. Min. Inst..

[42] Bemporad A. Model Predictive Control design: New trends and tools. Proceedings of the 45th IEEE Conference on Decision and Control.

[43] Zanoli S.M., Pepe C., Astolfi G. (2023). Advanced Process Control for Clinker Rotary Kiln and Grate Cooler. Sensors.

[44] César G.Q., Daniel S.H. (2009). Multivariable Model Predictive Control of a Simulated SAG plant. IFAC Proc. Vol..

[45] BrainWave SAG Mill. https://www.andritz.com/products-en/automation/advanced-process-control/brainwave-sag-mill.

[46] Chen S., Zhang T., Zou Y., Xiao M. (2019). Model predictive control of robotic grinding based on deep belief network. Complexity.

[47] Delaney G.W., Cleary P.W., Morrison R.D., Cummins S., Loveday B. (2013). Predicting breakage and the evolution of rock size and shape distributions in Ag and SAG mills using DEM. Miner. Eng..

[48] Saldaña M., Gálvez E., Navarra A., Toro N., Cisternas L.A. (2023). Optimization of the SAG Grinding Process Using Statistical Analysis and Machine Learning: A Case Study of the Chilean Copper Mining Industry. Materials.

[49] Bolobov V.I., Chupin S.A., Akhmerov E.V., Plaschinskiy V.A. (2021). Comparative Wear Resistance of Existing and Prospective Materials of Fast-Wearing Elements of Mining Equipment. Mater. Sci. Forum.

[50] Shishlyannikov D.I., Lavrenko S.A., Zverev V.Y., Muravskiy A.K., Mikryukov A.Y. (2023). Hydroabrasive wear of work stages of electric-centrifugal well pumps for fluids with high content of mechanical impurities. Min. Informational Anal. Bull..

[51] Serzhan S.L., Skrebnev V.I., Malevanny D.V. (2023). Study of the effects of steel and polymer pipe roughness on the pressure loss in tailings slurry hydrotransport. Obogashchenie Rud.

[52] Beloglazov I.I., Sabinin D.S., Nikolaev M.Y.U. (2022). Modeling the disintegration process for ball mills using dem. Min. Informational Anal. Bull..

[53] Boemer D., Carretta Y., Laugier M., Legrand N., Papeleux L., Boman R., Ponthot J.P. (2021). An advanced model of lubricated cold rolling with its comprehensive pilot mill validation. J. Mater. Process. Technol..

[54] Cleary P.W., Delaney G.W., Sinnott M.D., Morrison R.D. (2018). Inclusion of incremental damage breakage of particles and slurry rheology into a particle scale multiphase model of a SAG mill. Miner. Eng..

[55] Fedorova E.R., Vinogradova A.A. (2018). Generalized mathematical model of red muds’ thickener of alumina production. IOP Conf. Ser. Mater. Sci. Eng..

[56] Fedorova E.R., Pupysheva E.A., Morgunov V.V. (2023). Settling parameters determined during thickening and washing of red muds. Tsvetnye Met..

[57] Nikolaichuk L., Ignatiev K., Filatova I., Shabalovac A. (2023). Diversification of Portfolio of International Oil and Gas Assets using Cluster Analysis. Int. J. Eng..

[58] King R.P. (2001). Comminution operations. Modeling and Simulation of Mineral Processing Systems.

[59] Srivastava V., Akdogan G., Ghosh T., Ganguli R. (2018). Dynamic modeling and simulation of a SAG mill for mill charge characterization. Miner. Metall. Process..

[60] Kulchitskiy A. (2021). Optical Inspection Systems for Axisymmetric Parts with Spatial 2D Resolution. Symmetry.

[61] Global Mining Guidelines Group The Morrell Method to Determine the Efficiency of Industrial Grinding Circuits. https://gmggroup.org/guidelines-and-publications/morrell-method-to-determine-the-efficiency-of-industrial-grinding-circuits/.

[62] Nguyen V.T., Pham T.V., Rogachev M.K., Korobov G.Y., Parfenov D.V., Zhurkevich A.O.O., Islamov S.R. (2023). A comprehensive method for determining the dewaxing interval period in gas lift wells. J. Pet. Explor. Prod. Technol..

[63] Real-Time Estimation of SAG Mill Charge Characteristics for Process Optimization. https://www.researchgate.net/publication/374368429_Real-Time_Estimation_of_SAG_Mill_Charge_Characteristics_for_Process_Optimization.

